# SARS-CoV-2 and Pre-Tamponade Pericardial Effusion. Could Sotos Syndrome Be a Major Risk Factor?

**DOI:** 10.3390/genes12111782

**Published:** 2021-11-10

**Authors:** Barbara Citoni, Maria Cristina Digilio, Rossella Capolino, Maria Giulia Gagliardi, Andrea Campana, Fabrizio Drago, Giulio Calcagni

**Affiliations:** 1 Department of Pediatric Cardiology and Cardiac Surgery, Bambino Gesù Children’s Hospital, IRCCS, 00165 Rome, Italy; barbara.citoni@opbg.net (B.C.); mariagiulia.gagliardi@opbg.net (M.G.G.); fabrizio.drago@opbg.net (F.D.); 2 Genetics and Rare Diseases Research Division, Ospedale Pediatrico Bambino Gesù, IRCCS, 00165 Rome, Italy; mcristina.digilio@opbg.net (M.C.D.); rossella.capolino@opbg.net (R.C.); 3 Academic Department of Pediatrics, Bambino Gesù Children’s Hospital, IRCCS, 00165 Rome, Italy; andrea.campana@opbg.net

**Keywords:** SARS-CoV-2, COVID-19, Sotos syndrome, pericardial effusion, pericarditis

## Abstract

Pericarditis with pericardial effusion in SARS CoV-2 infection is a well-known entity in adults. In children and adolescents, only a few cases have been reported. Here, we present here a case of a 15-year-old girl affected by Sotos syndrome with pre-tamponed pericardial effusion occurred during SARS-CoV-2 infection. A possible relation between SARS-CoV-2 pericarditis and genetic syndromes, as a major risk factor for the development of severe inflammation, has been speculated. We emphasize the importance of active surveillance by echocardiograms when SARS-CoV-2 infection occurs in combination with a genetic condition.

## 1. Introduction

The clinical spectrum of Coronavirus Disease 19 (COVID-19) ranges from asymptomatic infections to multiorgan failure. The typical clinical presentation of COVID-19 is characterized by respiratory tract symptoms that include fever, cough, fatigue or dyspnea. However, the cardiovascular system has also been increasingly recognized as an important target of severe acute respiratory syndrome coronavirus 2 syndrome infection (SARS-CoV-2), leading to different diseases, such as acute coronary syndrome, cardiac arrhythmias, thromboembolism, myocarditis and pericarditis [1].

Data from China suggest that pediatric COVID-19 cases might be less severe than adult cases and that children might experience different symptoms compared to adults [2,3]. However, multi-inflammatory syndrome (MIS-C) may occur more frequently in children and adolescents than in the adult population. In this context, pericarditis and/or myocarditis have been previously reported as a possible consequence of this severe inflammatory disease [4,5].

Sotos syndrome is a genetic disorder characterized by childhood overgrowth, macrocephaly, developmental delays and a distinctive facial appearance caused by heterozygous mutation or deletion of the NSD1 gene [6,7]. Here, we describe a 15-years-old girl with Sotos syndrome presenting pre-tamponed pericardial effusion during SARS-CoV-2 infection, and speculate the possible predisposition role of this genetic disorder in the occurrence of more severe cardiac MIS-C.

## 2. Case Presentation

A 15-year-old girl affected by Sotos syndrome was admitted to our emergency department for fever, chest pain and SARS-CoV-2 infection. The girl is 175 cm (>95th percentile) tall and weighs 56.5 kg (62nd percentile). Clinical features included tall stature, macrocephaly, hypotonia and motor developmental delay, as well as facial anomalies (frontal bossing and triangular face with a pointed chin). She was surgically treated for intestinal atresia at birth, and residual minor tricuspid endocardial thrombus due to a previous septic central venous catheter was known and confirmed in previous echocardiograms performed during her clinical follow-up. Molecular analysis using target resequencing on a MiSeq (Illumina) platform showed a heterozygous c. 3585delT (P1195fsX1218) mutation in the *NSD1* gene.

**Figure 1:**
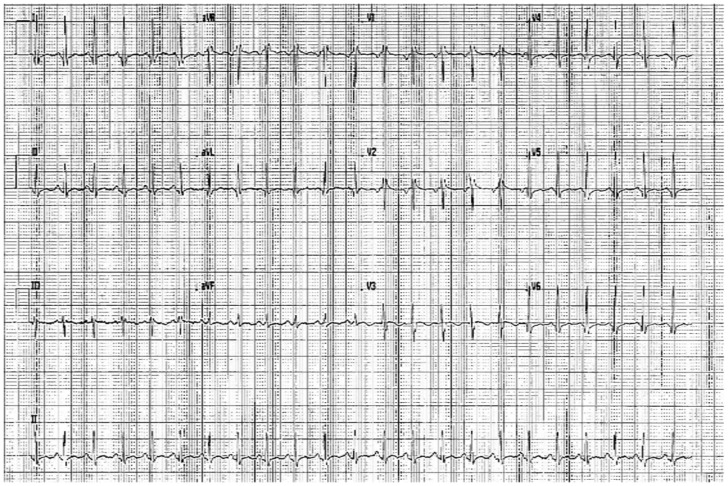
The 12-leads ECG performed at admission.

The girl had a fever a month before admission, which was treated with azithromycin and betamethasone for 6 days. Successively, SARS CoV-2 infection was confirmed by molecular nasopharyngeal swabs. At admission, she had dyspnea and chest pain. On physical examination: capillary filling <2 s, pale rosy skin, tachycardia, no significant murmurs, no rubbing and valid wrists. Body temperature was 37.6 °C, blood pressure was 110/65 mmHg, heart rate (HR) was 140 bpm, RR was 31 breaths per minute and oxygen saturation was 99%. Her electrocardiogram showed sinus tachycardia (HR 145 bpm), normal AV conduction, trivial right bundle branch block and aspecific repolarization abnormalities in the lower leads (Figure 1)

Blood tests, including hsTnI, were normal except C- reactive protein, which was 15.2 mg/dL (n.v. < 0.5 mg/dL), D-dimers 5.4 ng/mL (n.v. < 0.5 ng/mL), fibrinogen 849 mg/dL (n.v. range 212–433 mg/dL), NT-proBNP 312 pg/mL (normal value < 206 pg/mL), lactate dehydrogenase 524 U/L (n.v. range 135–225 U/L).

**Figure 2:**
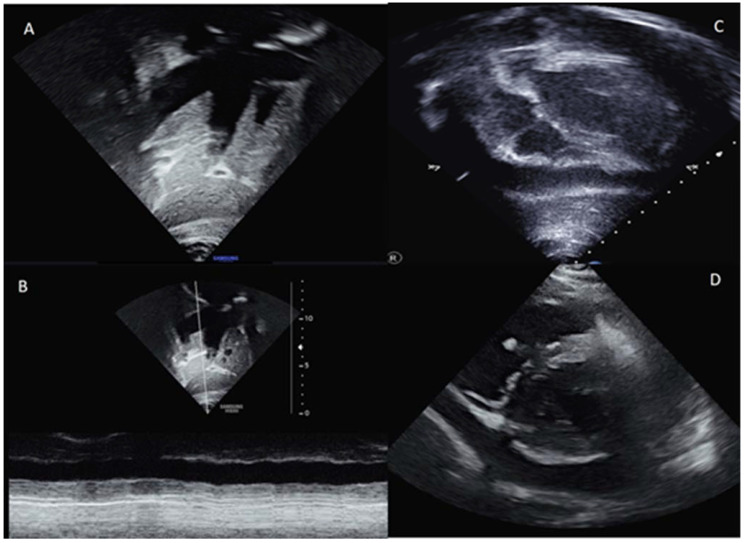
Echocardiogram images showing inferior vena cava and suprahepatic veins dilation (**A**); severe reduction of the normal inferior vena cava collapse (**B**); severe pericardial effusion in subxiphoid (**C**) and short axis view (**D**).

A chest X-ray showed a slight thickening of the peribronchovascular interstitium in the ilo-perilar region and at the base. Cardiac transthoracic ecocolorDoppler (Figure 2) revealed dilated inferior vena cava hypocollassing with respiratory acts. Severe ubiquitous pericardial effusion with signs of initial atrial collapse (maximum extent, especially in the lateral area of about 3 cm). There was good global biventricular function and no involvement of the coronary arteries was documented. Due to the clinical and hematological results, a suspicion of pediatric multisystem inflammatory syndrome (MIS-C) was raised, and she started treatment with anti-interleukin 1, intravenous immunoglobulins and corticosteroids.

Pericardiocentesis was performed in an emergency and pericardial drainage was maintained for 3 days. On pericardial effusion, fibrin, red blood cells and a fair share of leukocytes were documented, while microbiological tests excluded the presence of different virus such as Citomegalovirus, Ebstein Barr virus, Entheroviridae, Herpes Simplex Virus 1 and 2, Herpes Virus 6, Parvovirus B19 and SARS-CoV-2. All bacterial cultures were negative, and any viral infection was documented in the blood exams.

At discharge, after 14 days of recovery, steroid dosage was progressively reduced, anti interleukin1 therapy was suspended, while colchicine at a dosage of 0.5 mg/die was confirmed.

All blood exams were normal except for neutrophilic leucicotosis, hyperglyceridemia and hyperglemic, aspartate transaminase > 100 U/L (n.v. < 32 U/L), as well as alanine transaminase 158 U/L (n.v. < 33 U/L), which progressively reduced.

A few days after discharge, she was clinically evaluated and the absence of residual pericardial effusion and a good biventricular function were confirmed.

## 3. Discussion

We report the first pediatric patient with Sotos syndrome suffering from severe pericardial effusion during SARS-CoV-2 infection.

In the adult population, pericarditis with pericardial effusion related to COVID-19 infection has been reported [8]. Similarly, in the pediatric setting, only a few cases of pericarditis related to COVID-19 have been described. In one case, a seven-year-old female with pericardial tamponade, who was SARS-CoV-2-positive, underwent emergency pericardiocentesis followed by surgical pericardial decortication and pericardiectomy [9]. Non-clinical suspicion and/or genetic mutation were associated with these pediatric patients. Therefore, our patient is the first case reported in the literature in whom the occurrence of pericarditis with severe pericardial effusion, due to SARS-CoV-2 infection, coexists with the presence of a genetic condition.

An increased incidence of serositis and inflammatory manifestations can occur in specific genetic conditions, such as in patients with Familial Mediterranean fever (FMF). In these patients, a periodic fever may be associated with different serositis, arthritis, dermal manifestations and long-term renal complications. FMF is an autosomal recessive condition due to *MEFV* gene mutation, which encodes for an abnormal protein (pyrin), leading to an overexpressed inflammatory response secondary to an uncontrolled role of interleukin-1 [10]. Additionally, in patients with Turner syndrome, autoimmune disorders may lead to serositis [11]. Similarly, in RASopathies, a heterogeneous group of syndromes with common RAS MAPK cascade disorders, serositis, particularly after cardiac procedures, may occur. A possible association between RASopathies and autoimmune disorders has been suggested in a clinical and serological study of 42 patients [12]. This is due to the activation of the RAS/ MAPK pathway in immune cells that can contribute to the development of autoimmune diseases [13].

Sotos syndrome is a typical overgrowth syndrome presenting with advanced bone age, acromegalic features and a pointed chin, occasional brain anomalies and seizures and a nonprogressive cerebral disorder with mental retardation. Advanced bone age is present in approximately 75 to 85% of patients.

## 4. Conclusions

Our case represents the first patient presenting pericardial effusion related to COVID-19 infection in this condition. No unequivocal etiology of this disorder can be explained. A possible direct role of the SARS-CoV-2 virus in these patients, or an overexpressed inflammatory response, may be advocated. We would like to point out the importance of active surveillance in Sotos syndrome, considering that a worse pathogenic mechanism might be present. Experimental studies are also essential to investigate the pathogenetic mechanism generating an increased risk of serositis in patients with Sotos syndrome. This rare but possibly life-threatening association should always be considered, and clinicians should be aware of red flags regarding the management of this condition during SARS-CoV-2 infection so as to prevent the risk of major complications.
